# Type II collagen scaffolds repair critical-sized osteochondral defects under induced conditions of osteoarthritis in rat knee joints via inhibiting TGF-β-Smad1/5/8 signaling pathway

**DOI:** 10.1016/j.bioactmat.2024.02.008

**Published:** 2024-02-16

**Authors:** Xu Hu, Min Jin, Kang Sun, Zhen Zhang, Zhonglian Wu, Junli Shi, Peilai Liu, Hang Yao, Dong-An Wang

**Affiliations:** a Department of Biomedical Engineering, City University of Hong Kong, 83 Tat Chee Avenue, Kowloon, Hong Kong; b Karolinska Institutet Ming Wai Lau Centre for Reparative Medicine, HKSTP, Sha Tin, Hong Kong; c Department of Orthopedics, Qilu Hospital of Shandong University, 107 Wenhua Xilu, Jinan, PR China; d School of Chemistry and Chemical Engineering, Yangzhou University, Yangzhou, PR China; e Shenzhen Research Institute, City University of Hong Kong, Shenzhen, PR China

**Keywords:** Osteoarthritis, Osteochondral defect, Tissue engineering, Type II collagen

## Abstract

The bidirectional relationship between osteochondral defects (OCD) and osteoarthritis (OA), with each condition exacerbating the other, makes OCD regeneration in the presence of OA challenging. Type II collagen (Col2) is important in OCD regeneration and the management of OA, but its potential applications in cartilage tissue engineering are significantly limited. This study investigated the regeneration capacity of Col2 scaffolds in critical-sized OCDs under surgically induced OA conditions and explored the underlying mechanisms that promoted OCD regeneration. Furthermore, the repair potential of Col2 scaffolds was validated in over critical-sized OCD models. After 90 days or 150 days since scaffold implantation, complete healing was observed histologically in critical-sized OCD, evidenced by the excellent integration with surrounding native tissues. The newly formed tissue biochemically resembled adjacent natural tissue and exhibited comparable biomechanical properties. The regenerated OA tissue demonstrated lower expression of genes associated with cartilage degradation than native OA tissue but comparable expression of genes related to osteochondral anabolism compared with normal tissue. Additionally, transcriptome and proteome analysis revealed the hindrance of TGF-β-Smad1/5/8 in regenerated OA tissue. In conclusion, the engrafting of Col2 scaffolds led to the successful regeneration of critical-sized OCDs under surgically induced OA conditions by inhibiting the TGF-β-Smad1/5/8 signaling pathway.

## Highlights

• Osteochondral defects (OCDs) are prevalent without effective methods for complete regeneration.
• Regenerating OCD in the presence of osteoarthritis (OA) presents an even more significant challenge.
• Col2 scaffolds could repair critical-sized OCDs under OA conditions by inhibiting the TGF-β-Smad1/5/8 signaling pathway.
• The regeneration potential of Col2 scaffolds was validated in over critical-sized OCDs under the same condition.

## Graphical abstract

**Figure:**
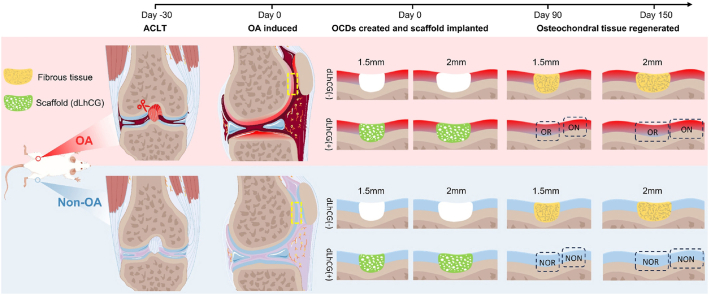

## 1 Introduction

Cartilage, particularly the hyaline cartilage in weight-bearing joints, exhibits minimal self-healing ability due to its avascularity and aneural nature [1]. The cells in cartilage rely on the diffusion of nutrients and oxygen from surrounding tissues for survival, making it challenging for them to self-repair and regenerate after an injury [1,2]. In addition, once significant discomfort arises, the defect typically permeates the non-sensitive nerve layer of the cartilage and invades the subchondral bone [3]. Therefore, the regeneration of osteochondral defects (OCDs) is a more prevalent and formidable challenge than the repair of cartilage injuries. Current treatments in clinical practice, such as microfracture, frequently result in the formation of mechanically inferior fibrocartilage in the long term [4]. Alternative therapies, like autologous chondrocyte implantation, are primarily limited by the availability of donor tissue and the requirement for a second surgery, which may inflict additional therapeutic trauma to the subchondral bone [4,5]. In short, OCDs are prevalent without effective methods for complete regeneration.

Osteoarthritis (OA) is a degenerative disease that causes the deterioration of articular cartilage, joint inflammation, synovitis, and subchondral bone changes [3]. In the presence of OA, increased inflammation may impair cartilage repair, while changes in the subchondral bone can negatively affect bone repair quality [6]. These factors suggest that OA may hinder the healing process of OCDs, a notion supported by clinical studies indicating that patients with early-stage OA exhibited better clinical outcomes than those with advanced-stage OA after intra-articular use of hyaluronic acid [7]. Therefore, the regeneration of OCDs in the presence of OA is much more challenging than in normal conditions.

For the OCD regeneration and the management of OA, type II collagen (Col2) is of crucial importance. Specifically, the regeneration of hyaline cartilage, which primarily consists of Col2, is essential for successful OCD repair, as the subchondral bone possesses inherent self-healing capabilities [1]. In the case of OA, inflammatory mediators such as cytokines and chemokines can degrade Col2 and impair cartilage function [8]. In addition, Col2 possesses a unique three-dimensional structure that facilitates its interaction with other components of the extracellular matrix, such as proteoglycans and glycosaminoglycans, to form a stable and functional chondro-inductive matrix [9]. The specific peptide sequences within Col2 enable its binding to receptors on the plasma membrane of mesenchymal stem cells, thereby activating intracellular signaling pathways that lead to the chondrogenic differentiation of cells [10,11]. Hence, Col2 can provide a more biomimetic microenvironment for chondrocyte differentiation, making it an ideal candidate for cartilage tissue engineering. However, the increased concentration of aspartic acid and glutamic acid residues in soluble Col2 interferes with the entropy-driven fibrillation process, resulting in the formation of less ordered fibrils and the collapse of scaffolds [12]. Although crosslinking reagents and polymer blending have been utilized to improve the mechanical integrity of Col2 scaffolds, the complex fabrication process and difficulties in achieving high levels of purity and yield have significantly limited the potential applications of Col2 scaffolds in cartilage tissue engineering [13].

**Fig. 1:**
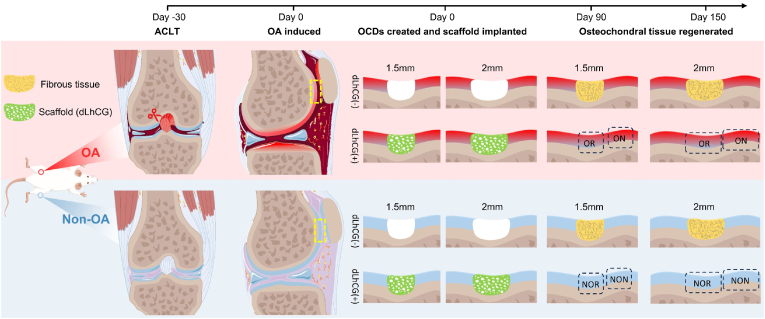
Schematic illustration of study design. Day −30: anterior cruciate ligament transection (ACLT) was performed on one hind limb (OA legs), leaving the other unoperated (non-OA legs). Day 0: osteoarthritis (OA) was induced; osteochondral defects (OCDs), measuring either 1.5 mm (critical-sized) or 2 mm (over critical-sized) in diameter, were created in both hind limbs in the non-weight-bearing area of the trochlear groove, followed by the engraftment of Col2 scaffolds; the 1.5 mm or 2 mm OCDs without scaffold implantation in the OA legs and non-OA legs served as negative control, referred to as OA dLhCG (−) and non-OA dLhCG (−), respectively. Day 90: 1.5 mm OCDs with dLhCG implantation were regenerated in OA and non-OA legs, while the 1.5 mm OCDs without dLhCG implantation were filled with fibrous tissue. Day 150: 2 mm OCDs treated with dLhCG were regenerated in OA and non-OA legs, while the 2 mm OCDs without dLhCG implantation were filled with fibrous tissue. In the OA legs, the osteochondral tissue regenerated from surgical sites and surrounding native tissue was referred to as 'OA regenerated' (OR) and 'OA native' (ON), respectively. In the non-OA legs, the osteochondral tissue regenerated from surgical sites and surrounding native tissue was referred to as 'non-OA regenerated' (NOR) and 'non-OA native' (NON), respectively.

To surmount these obstacles, we biosynthesized a pure hyaline cartilage matrix named dLhCG, which contained minimal type I collagen (Col1) [14]. Large animal studies have indicated that dLhCG engraftment in traumatic OCDs could induce fine regeneration of articular cartilage [14]. In this study, we aimed to investigate the regeneration capacity of Col2 scaffolds in critical-sized OCDs under surgically induced OA conditions and explore the underlying mechanisms that promoted OCD regeneration. Additionally, we sought to validate the regeneration potential of Col2 scaffold in over critical-sized OCDs under the same condition. The critical-sized OCD refers to the minimum size defect that cannot heal naturally and thus requires intervention to stimulate repair. In rats, a 1.5 mm-diameter defect in the trochlear groove of the knee joint is deemed a critical-sized OCD [15,16]. Recently, Katagiri et al. found that spontaneous regeneration did not occur in rat knees for OCDs that were 1.4 mm or greater in diameter, thus narrowing the value of critical-sized OCD to 1.4 mm [16]. To mimic the healing process of OCDs under surgically induced OA conditions, we first induced OA by performing anterior cruciate ligament transection (ACLT) in the rat knees. After 30 days since ACLT, critical-sized (1.5 mm diameter) or over critical-sized (2 mm diameter) OCDs were created in the non-weight-bearing areas of trochlear groove, followed by the engraftment of scaffolds (Fig. 1). Notably, the 2 mm-diameter OCD was approximately two times larger than the critical-sized defect. 90- or 150 days following scaffold implantation, the histological structure, biochemical composition, biomechanical function, transcriptome, and proteome of corresponding tissue were examined.

## 2 Materials and methods

### 2.1 The fabrication of a type II collagen scaffold

Unless explicitly stated, Sigma-Aldrich and Invitrogen, Life Technologies provided the chemicals and cell culture reagents. Col2 scaffolds were fabricated based on the previously established protocol (Supplemental Fig. 1) [14,17]. Briefly, passage 1 porcine chondrocytes (at a concentration of 10^7^ cells/ml) were combined with gelatin microspheres (150–180 μm diameter) and encapsulated in an alginate hydrogel. Cavities were subsequently created in the hydrogel by gelation dissolving at 37 °C in the incubator. The construct was cultivated in Dulbecco's Modified Eagle's Medium (DMEM) enriched with 20% fetal bovine serum (v/v), N-2-hydroxyethyl piperazine-N-2-ethane sulfonic acid (HEPES) at a concentration of 0.01 M, non-essential amino acids (NEAA) at 0.1 mM, l-proline at 0.4 mM, ascorbic acid at 0.05 mg/mL, vitamin C at 0.05 mg/mL, and penicillin-streptomycin at 100 units/mL for 35 days. During this period, chondrocytes proliferated within the alginate hydrogel and were guided to grow out of the gel phase, filling the voids and forming scattered pieces of pure microtissue. After removing the alginate by incubation with a 55 mM sodium citrate solution, the alginate-free constructs were cultured for an additional ten days. To produce the decellularized Col2 scaffold, the alginate-free constructs were subjected to three cycles of 3-h freezing at −80 °C and 4-h thawing at room temperature. Afterward, the constructs were soaked in a hypertonic TRIS solution for 24 h, followed by a hypotonic TRIS solution containing 1% Triton X-100 for 48 h. Finally, the constructs were immersed in DNase I (0.5 mg/mL) and RNase A (50 μg/mL) solutions for a duration of 3 h. The entire process was conducted on an orbital shaker with a speed of 150 rpm. The successful fabrication of Col2 scaffolds resulted in the production of a spongy, porous, and cartilaginous graft with a DNA content of less than 2 μg DNA per 1 mg dry weight [14].

### 2.2 Animal surgery

The Animal Research Ethics Committee of University reviewed and approved all animal surgeries and related experiments conducted in this study (Approval No. 202207206). This study involved the use of sixty-one male Sprague Dawley rats that were 12 weeks old. The experimental design of animal surgery was illustrated in Fig. 1, and the sample size for each experiment was detailed in Supplemental Table 1. In short, after OA induction, OCDs, measuring either 1.5 mm (critical-sized) or 2 mm (over critical-sized) in diameter, were created in OA and non-OA hind limbs, followed by the engraftment of Col2 scaffolds. Both traumatic OCDs invaded deeply into but did not extend beyond the subchondral bone, without the presence of other lesions such as cysts or tumors. Thus, this study involved four experimental conditions: non-OA legs +1.5 mm OCD, OA legs +1.5 mm OCD, non-OA legs +2 mm OCD, and OA legs +2 mm OCD. The 1.5 mm or 2 mm OCDs without scaffold implantation in the OA and non-OA legs served as negative control, referred to as OA dLhCG (−) and non-OA dLhCG (−), respectively. The preliminary experiment showed that the 1.5 mm or 2 mm OCDs with dLhCG implantation would regenerate on day 90 and day 150, respectively. In the OA legs, the osteochondral tissue regenerated from surgical sites and surrounding native tissue was referred to as 'OA regenerated' (OR) and 'OA native' (ON), respectively. In the non-OA legs, the osteochondral tissue regenerated from surgical sites and surrounding native tissue was referred to as 'non-OA regenerated' (NOR) and 'non-OA native' (NON), respectively.

### 2.3 Histological and immunohistochemistry staining

After euthanizing the rats, the distal femur bones of both legs were collected and fixed in 4% paraformaldehyde (PFA) for 48 h. The samples were then decalcified for 30 days in 10% ethylenediaminetetraacetic acid (EDTA) at room temperature. Subsequently, the samples were embedded in paraffin and sectioned in a sagittal direction at a thickness of 4 μm. Standard protocols were followed to perform histological staining with hematoxylin and eosin (H&E), safranin-o/fast green (Saf-O & FG), Mason's trichrome, and Picrosirius red [18]. The picrosirius red staining was imaged and photographed with an Olympus IX83 Inverted Microscope that supported polarized light observations. Immunohistochemistry staining was performed using the Immunohistochemistry (IHC) Detect Kit for Rabbit/Mouse Primary Antibody (Proteintech, PK10006). Shortly, deparaffinization and rehydration were performed, followed by antigen retrieval through either trypsin incubation (Thermo Scientific, 003009) for 30 min or heat-mediated antigen retrieval for 20 min. The slides were then blocked with 10% goat serum (Solarbio, SL038) at room temperature for 45 min, followed by 16-h incubation with primary antibodies, including Collagen II (ab34712), Collagen I (ab6308), and Osteocalcin (ab93876), at 4 °C. Endogenous peroxidase was quenched with 3% hydrogen peroxide for 20 min, and the secondary antibody was incubated for 30 min on the second day. Finally, DAB staining and counterstaining with hematoxylin were performed, and the slides were sealed with a DPX mounting medium (Sigma, 06522).

### 2.4 Microcomputed tomography

The distal femurs of both legs were fixed in 4% PFA for two days before the microcomputed tomography (micro-CT) test using the XtremeCT II (SCANCO Medical) system. All the samples were subjected to an X-ray with a voltage of 68 kVp and a beam current of 1464 μA to conduct a scan with a slice thickness of 20 μm. The Dragonfly 2022.2 (Object Research Systems Inc, Montreal, Canada) was used for image reconstruction, three-dimensional rendering, and bone morphological analysis. Cylindrical regions of interest were selected at the surgical site and surrounding the unoperated site, followed by the separation of subchondral cortical and trabecular bone. The parameters for trabecular bone microarchitecture, including bone volume fraction (BV/TV), trabecular thickness (Tb·Th), trabecular number (Tb·N), and trabecular separation (Tb.Sp), as well as parameters for cortical bone morphology, such as total cross-sectional area inside the periosteal envelope (Tt.Ar), average cortical thickness (Ct.Th), average cortical area (Ct.Ar), and cortical area fraction (Ct.Ar/Tt.Ar), were calculated [19].

### 2.5 Biochemical assays

The osteochondral tissue of interest was frozen at −20 °C, and the frozen samples were subjected to an overnight digestion process using papain solution (Solarbio, P8150). The content of DNA, glycosaminoglycans (GAGs), and hydroxyproline were quantified using the fluorometric Hoechst 33,258 assay (Invitrogen™, H3569), 1,9-dimethyl methylene blue (DMMB) assay, and l-hydroxyproline assay (acid hydrolysis method), respectively [[20], [21], [22]].

### 2.6 Biomechanical test

Following the extraction of fresh rat distal femurs, biomechanical tests were immediately conducted. The Young's modulus was analyzed using an unconfined setup on Instron equipment (USA), where the samples were kept in place at the patella groove. A compressive indenter was applied to the areas of interest, with a consistent speed of 200 μm per minute. The conditions for finishing the test were established as reaching 80% of the specimen's height or applying a compressive force of 30 N [17]. After obtaining a stress-strain curve, the elastic region of the curve was linearized to calculate Young's modulus of the tested tissue.

### 2.7 RNA sequencing

Six rats were euthanatized for RNA sequencing in 1.5 mm or 2 mm OCD models. For the 1.5 mm OCD model, regenerated osteochondral tissue at the surgical sites and surrounding native tissue in OA legs (OR and ON tissue) and non-OA legs (NOR and NON tissue) were harvested and fast-frozen in liquid nitrogen on day 90. This led to four groups: OR, ON, NOR, and NON. For the 2 mm OCD model, the corresponding tissue was collected on day 150. Due to the RNA scarcity of osteochondral tissue, two tissue pieces were pooled as a sample, resulting in three samples for every group in the 1.5 mm or 2 mm OCD model. BGI Tech Solutions Co., Ltd performed RNA extraction, library construction, and sequencing. Open-sourced software and programs, including fastp for quality control of raw data, HISAT2 for mapping sequencing reads, featureCounts for counting mapped reads, and DESeq2 for multigroup differential analysis of count data, were used for RNA sequencing analysis [[23], [24], [25], [26]]. A gene was deemed as having differential expression when its adjusted p-value was below 0.05, and the absolute value of its log2 fold change was higher than 1. Gene Ontology (GO) analysis and Gene Set Enrichment Analysis (GSEA) were adopted to identify the biological processes and pathways that were enriched with the genes of interest [27,28].

### 2.8 Mass spectrometry-based proteomics

The proteome of regenerated tissue and surrounding native tissue was evaluated by timsTOF Pro 2 (Bruker, United States) mass spectrometer in conjunction with data-independent acquisition parallel accumulation-serial fragmentation (dia-PASEF) method [29]. Nine rats were euthanatized for proteomics in 1.5 mm or 2 mm OCD models. For the 1.5 mm OCD model, regenerated osteochondral tissue at the surgical sites and surrounding native tissue in OA legs (OR and ON tissue) and non-OA legs (NOR and NON tissue) were harvested and fast-frozen in liquid nitrogen on day 90. This led to four groups: OR, ON, NOR, and NON. For the 2 mm OCD model, the corresponding tissue was collected on day 150. Three tissue pieces were pooled as a sample, resulting in three samples for every group in the 1.5 mm or 2 mm OCD model. The subsequent steps of tissue digestion, protein extraction, and proteomic data acquisition were carried out by BGI Tech Solutions Co., Ltd. Finally, the dia-PASEF data obtained were analyzed using FragPipe and DIA-NN [30]. ExpressVis web server was used for downstream visualization and analysis [31]. A protein was deemed as having differential expression when its adjusted p-value was below 0.15, and the absolute value of its log2 fold change was higher than 1.

### 2.9 Statistical analysis

All data were analyzed by SPSS 20.0 (SPSS Inc., USA) and presented as mean ± standard deviation. One-way analysis of variance (ANOVA) with a post hoc test was employed to detect the intergroup difference. Statistical significance was set at P < 0.05. In this manuscript, the significance levels with asterisks were denoted as follows: * indicated p < 0.05, ** indicated p < 0.01, and *** indicated p < 0.001.

## 3 Results

### 3.1 Histological, biochemical, and biomechanical analysis of critical-sized models

The histological results on day 90 (Supplemental Fig. 2) revealed the development of middle-to-late-stage OA in the legs treated with ACLT, while no significant changes were observed in the cartilage quality of either the femoral condyle or tibial plateau in the non-OA legs. In the OA leg, both the femur condyle and tibia plateau exhibited histopathological changes in the articular cartilage surface and subchondral bone, including disrupted and irregular cartilage surface, hypercellularity, a moderate decrease in Saf-O staining, and clefts that extended from the superficial to the deep zone and cartilage bone junction. In contrast, in the non-OA legs, the articular cartilage surface displayed a smooth articular cartilage texture with a normal distribution of chondrocytes and intact osteochondral junctions.

**Fig. 2:**
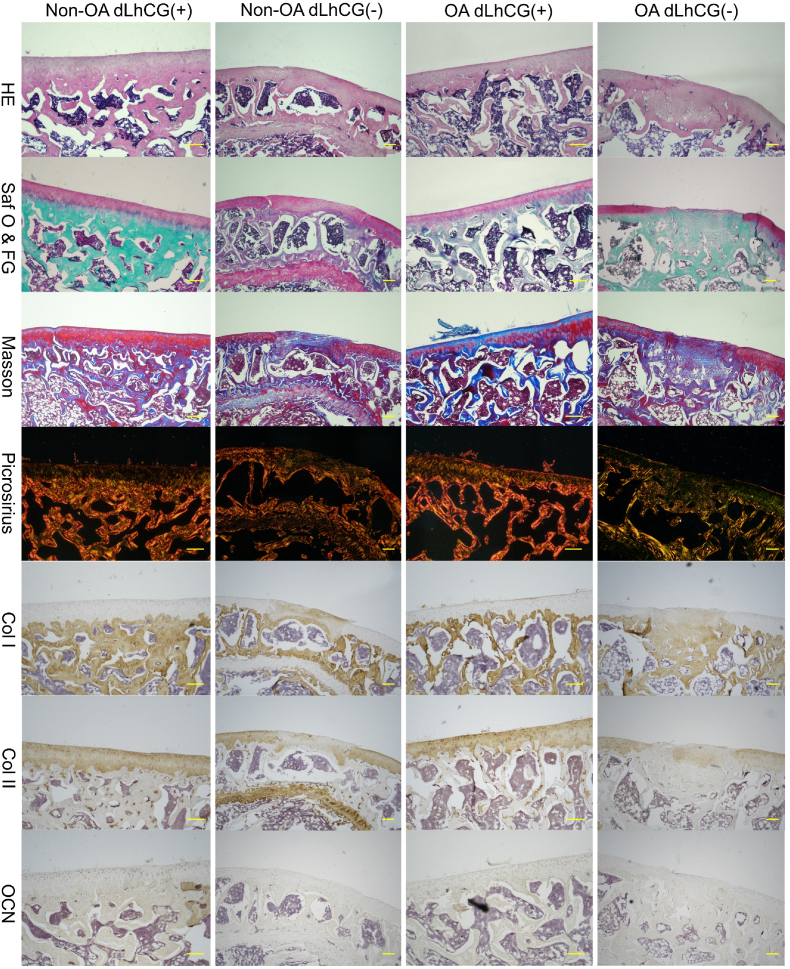
The histological staining of critical-sized osteochondral defects on day 90 revealed that non-OA and OA legs implanted with Col2 scaffolds exhibited complete osteochondral defect regeneration. In contrast, untreated samples demonstrated limited tissue regeneration, with either no regeneration or the most defective area filled with random fibrous tissue. Scale bar: 200 μm.

**Fig. 3:**
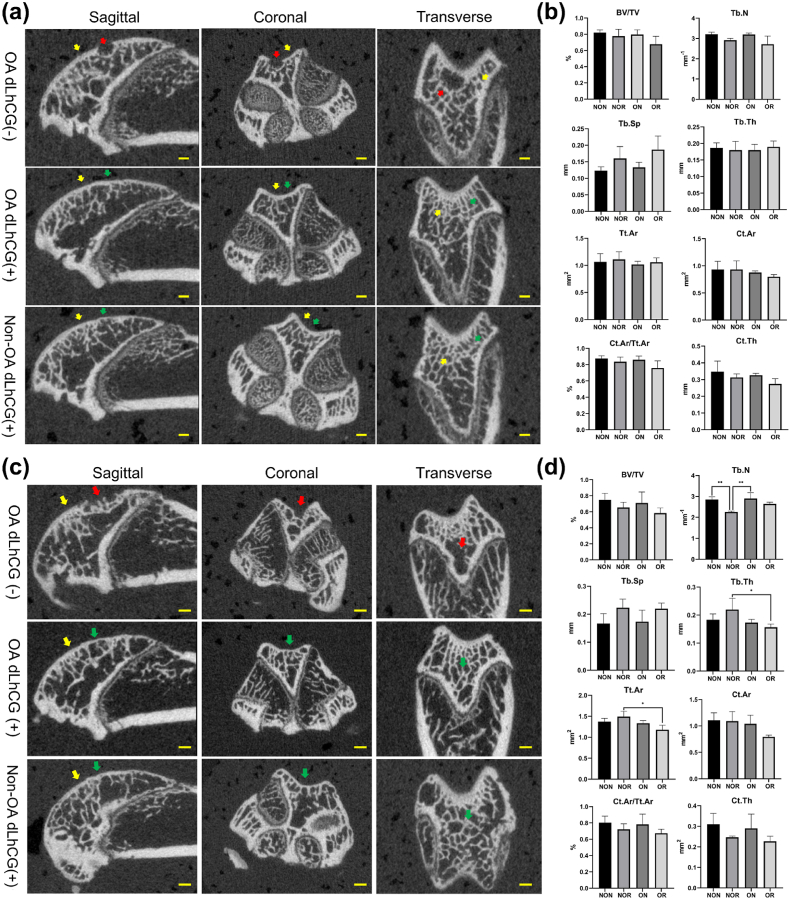
Microcomputed tomography. (a, b) Micro-CT images and bone morphological analysis of critical-sized OCDs on day 90. (c, d) Micro-CT images and bone morphological analysis of over critical-scale OCDs on day 150. Red arrow, untreated defect; yellow arrow, untreated native bone; green arrow, regenerated bone. OCD, osteochondral defects; BV/TV, bone volume fraction; Tb·N, trabecular number; Tb·Th, trabecular thickness; Tb.Sp, trabecular separation; Tt.Ar, total cross-sectional area inside the periosteal envelope; Ct.Ar, average cortical area; Ct.Ar/Tt.Ar, cortical area fraction; Ct.Th, average cortical thickness. NON, non-OA native; NOR, non-OA regenerated; ON, OA native; OR, OA regenerated. Scale bar: 1 mm.

As illustrated in Fig. 2, the histological staining for critical-sized OCDs on day 90 revealed that both non-OA and OA legs implanted with Col2 scaffolds (referred to as non-OA dLhCG (+) and OA dLhCG (+), respectively) exhibited complete regeneration of the OCD. Complete filling of OCDs with neo-cartilage on the surface and newly generated bony matrix in the underlying region was observed, with evidence of integration with surrounding native tissues. The cartilage layer was stained red with Saf-O & FG staining and tested positive for Collagen II. Meanwhile, the subchondral bone, which was stained green with Saf-O & FG staining, exhibited positive staining for both collagen I and osteocalcin. The Picrosirius staining allowed for the visualization of the regenerated tissue's ultrastructure. The surface layer of the newly formed cartilage and the adjacent natural cartilage appeared as a red line. In contrast, the middle and lower layers of the cartilage matrix were displayed roughly as yellow-green fibers oriented perpendicularly to the surface red line. Simultaneously, the subchondral bone was characterized as red or brown collagen fibers. On the contrary, the untreated samples (non-OA dLhCG (−) and OA dLhCG (−)) demonstrated limited tissue regeneration, with either no regeneration or the majority of the defective area filled with random fibrous tissue. Limited cartilage repair in untreated samples resulted in the discontinuity of the cartilage layer and a mixed phenotype of filling tissue. Micro-CT (Fig. 3a) further revealed the complete regeneration of subchondral bone in the samples implanted with Col2 scaffolds in either the OA or non-OA legs. No discernible void spaces were observed in the regenerated bone (indicated by the green arrow), which demonstrated no significant difference in comparison to the adjacent native bone (marked by the yellow arrow). Conversely, untreated defects exhibited substantial void spaces in sagittal, coronal, and transverse planes. Additionally, there were no significant differences in bone morphological parameters among the tissue samples obtained from 4 distinct locations (Fig. 3b).

**Fig. 4:**
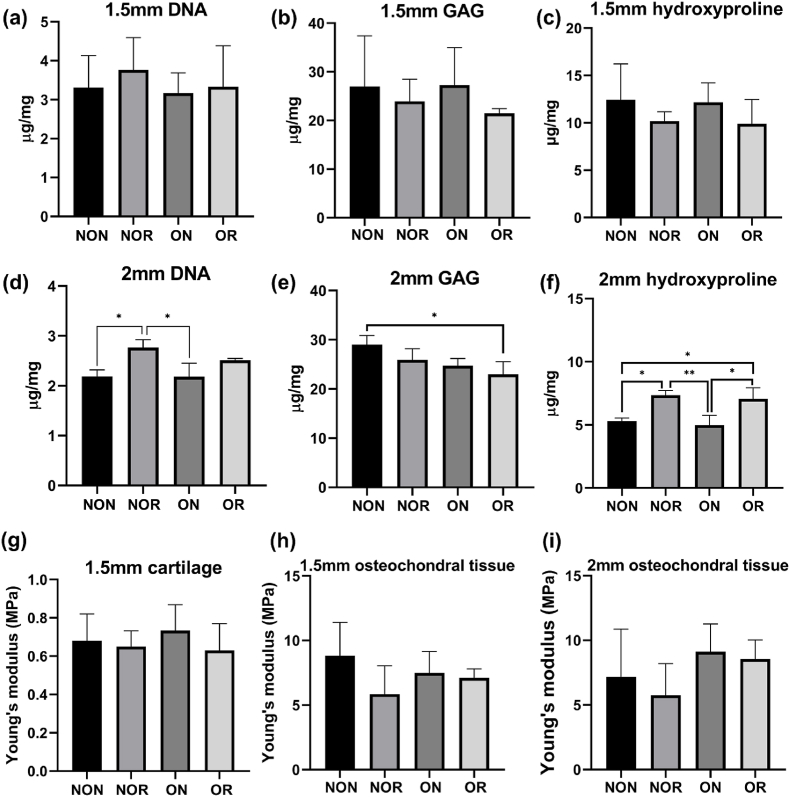
(a–c) The quantification results of DNA, glycosaminoglycans (GAGs), and hydroxyproline for critical-sized osteochondral defects (OCDs) on day 90. (d–f) The quantification results of DNA, GAGs, and hydroxyproline for over critical-sized OCDs on day 150. (g, h) Biomechanical analysis of critical-sized OCDs on day 90. (i) Biomechanical analysis of over critical-sized OCDs on day 150. NON, non-OA native; NOR, non-OA regenerated; ON, OA native; OR, OA regenerated.

In line with the bone morphology results, the biochemical analysis of 1.5 mm OCD found no significant differences among tissue samples from 4 locations (Fig. 4a, 4b, 4c). It was noteworthy that irrespective of whether it was a 1.5 mm or 2 mm OCD model, a trend was observed where the NOR and OR tissue exhibited higher or significantly higher DNA content than their corresponding natural tissues (Fig. 4a and 4d). These findings indicated that the regenerated tissue exhibited a greater cellular density compared to the adjacent native tissue, suggesting an ongoing recellularization process during the course of regeneration. Interestingly, regardless of whether it was a 1.5 mm or 2 mm OCD model, no significant difference in GAG content was observed between the regenerated tissue and the surrounding native tissue, even though the GAG content of NON tissue was found to be significantly higher than OR in 2 mm OCD model (Fig. 4e). Given that GAG predominantly exists in cartilage, the histological and GAG results indicated that cartilage regeneration was achieved under both normal and osteoarthritic conditions in critical-sized models through the implantation of Col2 scaffolds.

It was observed that under identical experimental conditions, the pattern of the stress-strain curve varied depending on the size of OCDs. Notably, the stress-strain curve of the tissue from over critical-sized OCDs demonstrated a linear pattern, while that for critical-sized OCDs exhibited a discontinuous pattern (Supplemental Fig. 3). The initial segment of the discontinuous curve had a relatively small slope, which significantly increased in the latter segment. This finding was likely attributable to the anatomical structure of osteochondral tissue, where the cartilage layer is located above the subchondral bone. Thus, it was plausible to infer that the initial segment of the stress-strain curve represented the mechanical characteristics of the cartilage, whereas the latter segment reflected the mechanical performance of the entire osteochondral tissue. Despite the differences between cartilage and entire osteochondral tissue, both the regenerated and the surrounding native tissue demonstrated comparable Young's modulus in critical-sized OCDs (Fig. 4g and 4h), suggesting that the regenerated tissue was capable of functioning similarly to the native tissue.

### 3.2 Histological, biochemical, and biomechanical analysis of over critical-sized models

**Fig. 5:**
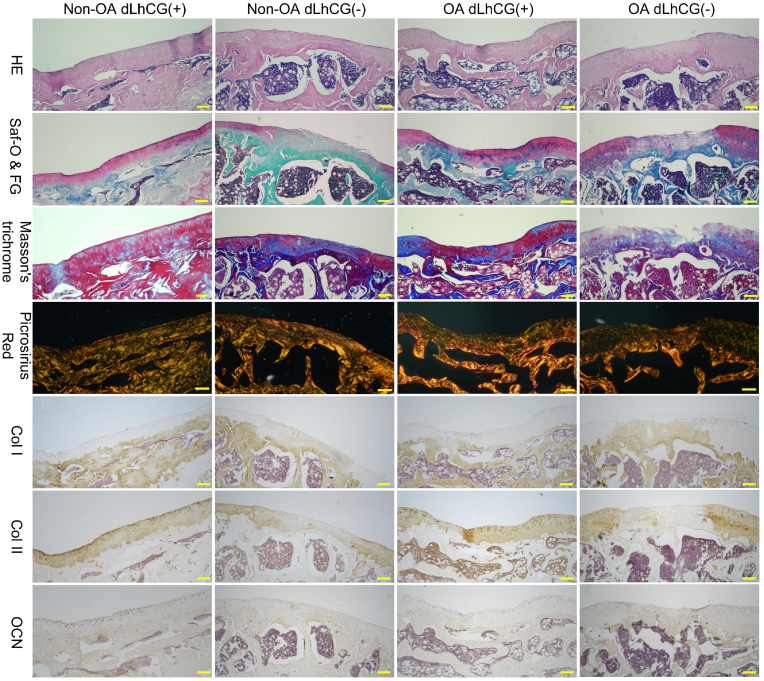
The histological staining for over critical-sized osteochondral defects on day 150 revealed that the samples engrafted with dLhCG exhibited satisfactory osteochondral repair, albeit with minor imperfections. In contrast, non-OA dLhCG (−) and OA dLhCG (−) samples exhibited sparse and random fibrous tissue infiltration. Scale bar: 200 μm.

The osteochondral regeneration was further validated in an over critical-sized OCD model under the same surgically induced OA condition. For the regeneration process of 2 mm OCD, there was a partial repair on day 80 and day 100 following Col2 scaffold implantation (shown in Supplementary Fig. 4 and Supplementary Fig. 5, respectively). At the 150-day postoperative evaluation, the samples engrafted with Col2 scaffolds exhibited satisfactory osteochondral repair, albeit with minor imperfections (Fig. 5). The cartilage layer exhibited an abundance of hyaline cartilage tissue, which was identified by positive staining for Col2, negative staining for Col1, and red coloration when subjected to Saf-O & FG staining. However, certain surface areas of the cartilage layer exhibited irregularities. Notably, in the OA dLhCG (+) sample, bone-like tissue occurred in the middle and deep cartilage layer, which displayed negative staining for Col2, positive staining for Col1, and appeared green with Saf-O & FG staining. In contrast, non-OA dLhCG (−) and OA dLhCG (−) samples exhibited sparse and random fibrous tissue infiltration.

On the other hand, the representative micro-CT images of 2 mm OCD on day 150 (Fig. 3c) were similar to those of 1.5 mm OCD on day 90 (Fig. 3a). The samples implanted with Col2 scaffolds demonstrated integral bone regeneration, whereas the OCDs without scaffold implantation displayed extensive gaps. Regarding parameters concerning trabecular bone microarchitecture (Fig. 3d), there were no significant differences in BV/TV and Tb.Sp among tissue samples from 4 different locations. Nonetheless, the NOR tissue exhibited distinct bone microarchitecture features compared to the NON, as evidenced by the significantly lower value for Tb·N but higher values for Tb·Th and Tt.Ar. Regarding cortical bone morphology, no significant differences in Ct.Ar, Ct.Ar/Tt.Ar, and Ct.Th were observed among four types of tissue. Regardless of whether it was a 1.5 mm or 2 mm OCD model, no significant differences were observed in any of the analyzed parameters between the ON and OR tissue, suggesting that Col2 scaffolds could be an effective method for repairing subchondral bone in critical-sized or over critical-sized OCDs within the context of OA. Notably, OA-affected subchondral bones (ON and OR tissue) did not exhibit the morphological changes reported in some OA animal models, such as increased Tb.Sp, decreased bone volume fraction or reduced Tb·Th [32]. The discrepancy between the results of this study and those of others may stem from differences in the choice of anatomical location for bone morphology analysis. Specifically, our study focused on a non-weight-bearing area of the knee with relatively mild symptoms of OA, whereas other studies selected weight-bearing areas such as the tibial plateau or femoral condyle with much more severe OA symptoms.

Previous studies identified the Tb·N and Tb.Sp were the two most important predictors of Young's modulus in human vertebrae and femur [33]. In the 2 mm OCD model, the NOR tissue, which was characterized by the lowest Tb·N but the highest Tb.Sp, demonstrated the lowest Young's modulus values in the biomechanical test (Fig. 3, Fig. 4i). However, no significant differences in Young's modulus were observed among the tissue from 4 distinct locations, with the highest values of Young's modulus in the ON, followed by the OR.

Collectively, Col2 scaffolds could effectively regenerate critical-sized and over critical-sized OCDs under a surgically induced OA condition, with the newly formed tissue histologically and biochemically resembling adjacent natural tissue and exhibiting comparable biomechanical properties.

### 3.3 Transcriptome and proteome analysis of critical-sized models

**Fig. 6:**
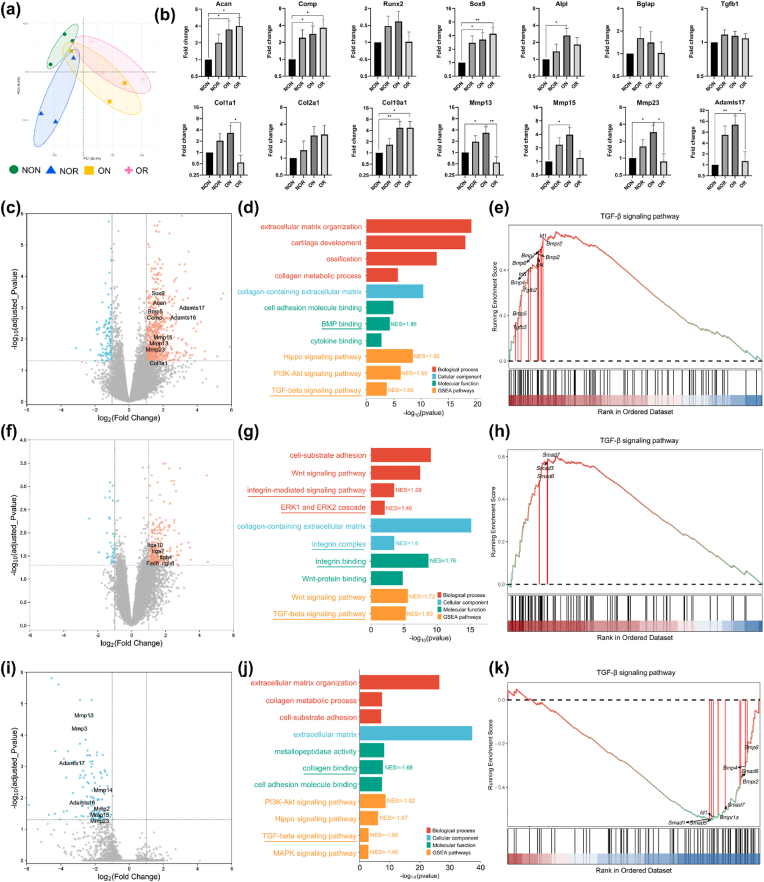
Gene expression analysis of the critical-sized osteochondral defects on day 90. (a) Principal component analysis. (b) Relative expression level of key genes. (c, d, e) Volcano plot, gene enrichment analysis, and GSEA of TGF-β signaling pathway for ON vs. NON. (f, g, h) Volcano plot, gene enrichment analysis, and GSEA of TGF-β signaling pathway for NOR vs. NON. (i, j, k) Volcano plot, gene enrichment analysis, and GSEA of TGF-β signaling pathway for OR vs. ON. GSEA, gene set enrichment analysis; NES, normalized enrichment score; NON, non-OA native; NOR, non-OA regenerated; ON, OA native; OR, OA regenerated.

**Fig. 7:**
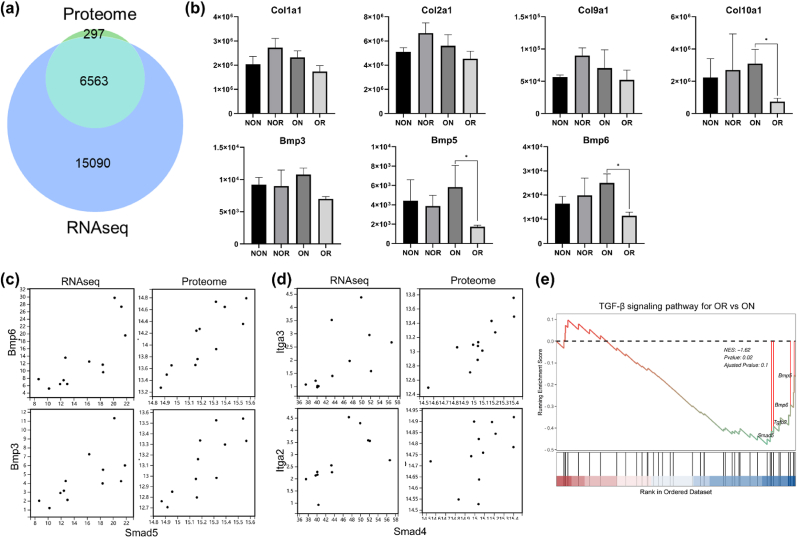
The proteome analysis of the critical-sized osteochondral defects on day 90. (a) Venn diagram for proteins in proteome and genes in the transcriptome. (b) Relative protein quantification across the tissue samples. (c, d) The integration analysis of transcriptome and proteome. (e) Gene set enrichment analysis of TGF-β signaling pathway for OR vs. ON. NES, normalized enrichment score; NON, non-OA native; NOR, non-OA regenerated; ON, OA native; OR, OA regenerated.

The gene expression analysis of the critical-sized model identified a total of 21,653 genes across the tissue samples from 4 distinct locations. The general gene expression pattern of four types of tissue was revealed by principal component analysis (PCA, Fig. 6a). In PCA, samples located far apart are likely to have distinct gene expression profiles, representing different experimental conditions or treatments [34]. It could be observed that NOR, ON, and OR tissue overlapped with each other, while NON tissue was separated from the NOR and OR. When probing into the specific gene expression levels and setting NON tissue as reference (Fig. 6b), it was highlighted that the ON tissue demonstrated the highest or significantly higher expression levels of genes related to osteochondral anabolism, including Acan (aggrecan), Comp (cartilage oligomeric matrix protein), Runx2, Sox9, and Col2a1, Alpl (alkaline phosphatase), Tgfb1 (transforming growth factor beta 1), Col1a1, and Col2a1. Meanwhile, the ON tissue exhibited the highest expression of genes related to cartilage degradation, such as Mmp13 (matrix metallopeptidase), Mmp15, Mmp23, and Adamts17 (a disintegrin and metalloproteinase with thrombospondin motifs). It seemed that the ON tissue exhibited significantly higher gene expression associated with osteochondral anabolism and cartilage degradation while also displaying the second-highest value for hydroxyproline and GAG mass (Fig. 4b and 4c). This observation suggested an equilibrium between the synthesis and breakdown processes in the ON tissue, resulting in a comparable hydroxyproline and GAG quantity to that of the NON tissue. Moreover, Col10a1, primarily expressed in hypertrophic chondrocytes, was significantly upregulated in ON tissue. The significant upregulation of Col10a1 gene expression was confirmed through relative protein quantification in the following proteome analysis (Fig. 7b). These findings provided evidence of cartilage destruction and inflammation in the joint cavity affected by OA, thereby confirming the successful establishment of the OA model.

The volcano plot of ON vs. NON (Fig. 6c) confirmed the presence of several significantly upregulated genes in the ON tissue, including Bmp5, Acan, Comp, Sox9, Mmp13, Mmp15, Mmp23, Adamts16, Adamts17, Col1a1, etc. The GSEA revealed that Hippo, PI3K-Akt, and TGF-β/Bmp signaling pathways significantly upregulated in the ON tissue (Fig. 6d), all of which have been reported to be tightly associated with cartilage and OA [[35], [36], [37], [38]]. The Hippo signaling played a crucial role in regulating the natural functions of cartilage [35,36]. Activation of the PI3K-Akt pathway led to increased Mmp production, while inhibition of this pathway alleviated post-traumatic OA [37,38]. Of particular importance was the TGF-β signaling pathway, as the TGF-β superfamily, including TGF-βs, Activin, bone morphogenetic proteins (BMPs), and other related proteins, has a dual role in maintaining cartilage homeostasis. The switch from TGF-β-Smad 2/3 to BMP-Smad1/5/8 signaling contributed to osteophyte formation and the pathogenesis of OA [39,40]. In the comparison between ON and NON tissue, GO analysis (Fig. 6d) further demonstrated that the differentially expressed genes were significantly enriched in response to BMP and BMP binding. Additionally, GESA (Fig. 6e) verified the activation of the TGF-β/Bmp-Smad1/5/8 signaling pathway in ON tissue, with several critical genes in this pathway, such as Bmp5, Bmp6, Bmp 7, Smad1, Smad5, and Id4, found to be upregulated.

On the other hand, when comparing NOR tissue with NON, significant upregulation of Itga10 (integrin subunit alpha 10), Itga7, Itga4, and Itga8 was observed in the volcano plot (Fig. 6f). The upregulated genes primarily participated in the ERK1/ERK2 cascade, integrin complex, and integrin binding (Fig. 6g). These findings aligned with the study of Lian et al. in which Col2a1 was found to interact with integrin, leading to ERK1/2 phosphorylation and subsequent repression of Smad1 activation, thus inhibiting BMP-Smad1/5/8-mediated chondrocyte hypertrophy. GESA validated the activation of the TGF-β/Bmp-Smad 2/3 signaling pathway and upregulation of Smad 3, Smad 6, and Smad 7 in ON tissue (Fig. 6h). Finally, the intergroup comparison between OR vs. ON found that several genes related to cartilage degradation (Mmp3, Mmp13, Adamts16, Adamts17, etc.) were significantly downregulated (Fig. 6i), indicating that OR tissue experienced much less cartilage and extracellular matrix breakdown than ON. In contrast to the comparative analysis between ON and NON, several pathways associated with inflammation and OA, such as Hippo, PI3K-Akt, and MAPK signaling pathways, were found to be downregulated in OR tissue (Fig. 6j). Specifically, GESA provided additional evidence supporting the inhibition of the TGF-β/Bmp-Smad1/5/8 pathway in OR tissue, with critical genes in this pathway, such as Bmpr1a, Smad1, Smad5, and Id1, being downregulated (Fig. 6k).

**Fig. 8:**
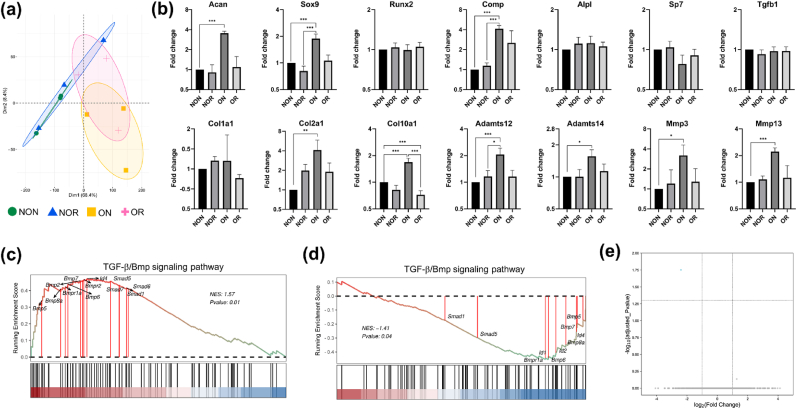
Gene expression analysis of the over critical-sized osteochondral defects on day 150. (a) Principal component analysis. (b) Relative expression level of key genes. (c) GSEA of TGF-β signaling pathway for ON vs. NON. (d) GSEA of TGF-β signaling pathway for OR vs. ON. (e) Volcano plot for NOR vs. NON. GSEA, gene set enrichment analysis; NES, normalized enrichment score; NON, non-OA native; NOR, non-OA regenerated; ON, OA native; OR, OA regenerated.

Proteome analysis of critical-sized OCDs evaluated the protein content across the tissue samples from 4 distinct locations. A total of 6860 proteins were detected, and 6563 of these had the same gene symbols with RNA sequencing (Fig. 7a). The relative protein quantification analysis (Fig. 7b) revealed no significant difference in the protein content of Col1a1 and Col2a1 across four types of tissue. However, the NOR tissue contained over 1.5 times more protein content of Col9a1 (a protein primarily found in neo-cartilage) than NON, suggesting the rejuvenation of regenerated tissue under normal conditions. Furthermore, ON tissue had more protein content of Bmp 3, Bmp5, and Bmp6 than OR. The integration analysis of transcriptome and proteome (Fig. 7, Fig. 8d) demonstrated that Bmp5 and Bmp6 were tightly correlated with Smad5, while Itga3 and Itga2 were closely associated with Smad 4, indicating the involvement of Bmp and Itga proteins in TGF-β/Bmp-Smad1/5/8 and TGF-β-Smad 2/3 signaling pathways, respectively. Finally, the GSEA of proteome supported the transcriptome analysis, revealing that TGF-β/Bmp-Smad1/5/8 was hindered in OR tissue compared with ON, with key genes in this pathway, such as Smad5, Bmp5, and Bmp6, being downregulated (Fig. 7e).

### 3.4 Transcriptome analysis of over critical-sized models

Similar to the findings of critical-sized OCD, the gene expression analysis of over critical-sized OCD revealed a distinct expression pattern of OR tissue (Fig. 8a). Specifically, the expression of genes associated with cartilage degradation, such as Adamts12, Adamts14, Mmp3, and Mmp13, were lower in OR than in ON tissue, while the genes related to osteochondral anabolism, including Acan, Sox9, Runx2, Comp, Alpl, Sp7, and Tgfb1, were expressed comparably between OR and NON tissue (Fig. 8b). Additionally, GSEA indicated that the TGF-β/Bmp-Smad1/5/8 signaling pathway was activated in ON tissue but inhibited in OR tissue (Fig. 8c and 8d), which was consistent with the enrichment analysis of critical-sized OCD. In contrast, only one differentially expressed gene was detected when comparing NOR tissue with NON (Fig. 8e), which was in agreement with the gene expression patterns revealed by the PCA plot where the confidence ellipse of NOR tissue almost encircled that of NON (Fig. 8a). These findings suggested that the gene expression of regenerated tissue closely resembled that of the surrounding natural tissue even in over critical-sized OCDs.

Collectively, the study proved that the engrafting of Col2 scaffolds led to the successful regeneration of critical-sized OCDs under a surgically induced OA condition by inhibiting the TGF-β-Smad1/5/8 signaling pathway. Furthermore, the repair potential of Col2 scaffolds was validated in over critical-sized OCD models.

## 4 Discussion

OCDs, especially when associated with OA, can lead to persistent pain, inflammation, and reduced joint function. If left untreated, OCDs significantly reduce the quality of life and lead to long-term disability [3,6]. Regenerative scaffold procedures, including cell-free and cell-based scaffolds, are emerging as potential therapeutic options for the treatment of OCD. Cell-free scaffolds, in particular, are gaining popularity due to their lower cost, reduced regulatory hurdles, simplified manufacturing process, and ease of surgical procedures [41].

Col2 is the characteristic component of articular cartilage and is considered an ideal material for cartilage repair scaffolds because of its biocompatibility, structural similarity, cell interaction, and biodegradability [42]. Col2 allows for the creation of engineered scaffolds that closely resemble the composition of the extracellular matrix of cartilage. In comparison to Col1, Col2 scaffolds have demonstrated superior chondrogenic ability [[43], [44], [45]]. In vitro studies revealed that Col2 scaffolds maintained the round morphology of chondrocytes, significantly increased the synthesis of GAGs, and enhanced chondrogenic gene expression of stem cells more effectively than Col1 [[43], [44], [45]]. Additionally, in vivo experiments showed that Col2 scaffolds resulted in statistically higher cartilage repair scores, more effectively induced chondrocyte phenotype, and regenerated more hyaline cartilaginous tissue compared with Col2 [[46], [47], [48]]. Furthermore, preclinical studies have found that Col2 devices promoted efficient defect filling and hyaline neocartilage formation, highlighting their potential for clinical use [14]. In contrast, clinical studies have revealed the ineffectiveness of Col1 scaffolds in regenerating OCD and large cartilage defects in the long run. Applying a cell-free Col1 scaffold for extensive cartilage defects demonstrated an escalated wear of the repair tissue and a clinical failure rate of 18% at 5-year follow-up [49]. Similarly, implanting a biomimetic scaffold consisting of Col1 and hydroxyapatite resulted in incomplete cartilage regeneration and poor subchondral bone repair at 1- to 2.5-year follow-up [50].

The superior effectiveness of Col2 in cartilage regeneration may be attributed to its ligands or other bounded cartilage-specific constituents that can trigger specific biochemical signals for cell recruitment, chondrogenic differentiation, and homeostasis. Col2 has been found to mitigate chondrocyte hypertrophy and slow down the progression of OA by facilitating interaction with integrin β1 and competing with BMP receptors for binding to Smad1 [39]. Furthermore, the unique ultrastructure of Col2 allows for the intercalation of numerous proteoglycans, thus promoting extracellular matrix production and ensuring the water-binding capacity and elasticity of regenerated cartilage tissue [51]. Despite concerns regarding the induction of inflammatory arthritis in rats upon exposure to native Col2 from other species and the presence of antibodies against Col2 in patients with arthritis [52,53], it was necessary to note that effectively crosslinked Col2 did not induce arthritis in rats [54].

The current study represented the first attempt to investigate the repair efficiency of Col2 scaffolds under OA conditions and to investigate the underlying mechanisms that promoted OCD regeneration. Although the pivotal role of Col2 in chondrogenesis and cartilage regeneration is well-documented, the availability of high-purity Col2 remains limited [42,55]. To the best of our knowledge, only a few companies offer pure Col2, while the production of Col2 scaffolds is only feasible in small quantities in laboratory settings, rendering them impractical for widespread application. An alternative approach that has been explored for decades is the direct decellularization of natural cartilage tissue to obtain cartilaginous extracellular matrix, with BioCartilage (Arthrex) being a representative product in the commercial market [56]. However, preparing a decellularized matrix from hard tissue confronts the inherent dilemma that maintaining mechanical strength/structural integrity and eliminating immunogenicity cannot be achieved simultaneously [51]. The extensive removal of cellular components and extracellular matrix results in a loss of mechanical stability or the collapse of constructs, while leaving residual components can trigger an immune response in the recipient. On the contrary, our tissue-engineered cartilaginous graft was decellularized from a porous and soft hyaline cartilage matrix synthesized by chondrocytes [14], as illustrated in Supplementary Fig. 1. In contrast to the direct decellularization process from hard tissue, which involves strong acid or alkali solution and protease, the fabrication of dLhCG is achieved in a much milder manner, allowing for the preservation of crucial ligands or moieties that can promote oriented cell division, adhesion, and proliferation. This inference was validated by the proteomics analysis of dLhCG (Supplementary Fig. 7), which identified the presence of type 2 collagen, as well as various glycoproteins (aggrecan core protein, glypican, syndecan, hyaluronan, and proteoglycan link protein 1), extracellular matrix proteins (integrin, laminin), protein Wnt, and TGF-β superfamily.

The relationship between OA and OCDs is bidirectional, with each condition exacerbating the other. On the one hand, persistent OCDs result in inflammation, the release of enzymes that break down the cartilage, and mechanical instability of the joint, ultimately leading to the development of OA [57]. On the other hand, OA could cause cartilage loss and increased stress on the underlying bone, contributing to the deterioration of OCDs, particularly in weight-bearing areas. In addition, the cytokines and chemokines in the OA environment make the OCDs more challenging to heal [3,6]. In this study, to simulate the repair process of OCDs under the influence of the inflammatory environment of OA, we first induced knee OA using anterior cruciate ligament transection and subsequently created critical-sized OCDs in the non-weight-bearing area of the trochlear groove. It was believed that OA in weight-bearing regions would lead to the accumulation of synovial fluid rich in inflammatory cytokines and enzymes, thereby creating a significant OA-like environment throughout the joint cavity [8]. As stated previously, dLhCG possesses crucial ligands or moieties that facilitate osteochondral regeneration. The current study demonstrated that Col2 scaffolds could regenerate critical-sized OCDs under OA conditions by inhibiting the TGF-β-Smad1/5/8 signaling pathway. Thus, it could be inferred that Col2 scaffolds (dLhCG) can break the vicious circle of OCD and OA by effectively regenerating OCDs in the presence of OA, ultimately leading to the alleviation of OA.

## 5 Conclusion

For the first time, this study demonstrated that the implantation of Col2 scaffolds could repair critical-sized OCDs under surgically induced OA conditions through the inhibition of the TGF-β-Smad1/5/8 signaling pathway. After 90 days since the scaffold implantation, complete healing was observed histologically in critical-sized OCD, with evidence of excellent integration with surrounding native tissues. The newly formed tissue biochemically resembled adjacent natural tissue and exhibited comparable biomechanical properties. Furthermore, the regeneration potential of Col2 scaffolds was validated in over critical-sized OCDs under the same condition, with promising histological results observed after 150 days since scaffold placement. Gene expression analysis revealed a distinct expression pattern of OR tissue, with lower expression of genes associated with cartilage degradation than surrounding ON tissue but comparable expression of genes related to osteochondral anabolism compared with normal tissue. Additionally, transcriptome and proteome analysis demonstrated the hindrance of TGF-β-Smad1/5/8 in regenerated OA tissue, which was consistent with the transcriptome analysis in over critical-sized OCDs. In conclusion, the engrafting of Col2 scaffolds led to the successful regeneration of critical-sized OCDs under a surgically induced OA condition by inhibiting the TGF-β-Smad1/5/8 signaling pathway.

## Ethics approval

The Animal Research Ethics Committee of Yangzhou University reviewed and approved all animal surgeries and related experiments conducted in this study (Approval No. 202207206).

## Consent to participate

All authors consent to participate in this submission.

## CRediT authorship contribution statement

**Xu Hu:** Writing – review & editing, Writing – original draft, Visualization, Validation, Methodology, Investigation, Formal analysis, Data curation. **Min Jin:** Validation, Software, Methodology. **Kang Sun:** Validation, Software. **Zhen Zhang:** Validation, Software. **Zhonglian Wu:** Methodology, Software. **Junli Shi:** Methodology, Software. **Peilai Liu:** Writing – review & editing, Resources, Conceptualization. **Hang Yao:** Writing – review & editing, Resources, Project administration, Funding acquisition. **Dong-An Wang:** Writing – review & editing, Supervision, Resources, Funding acquisition, Conceptualization.

## Declaration of competing interest

The authors declare that they have no known competing financial interests or personal relationships that could have appeared to influence the work reported in this paper.
