# Supplementary material for: Type II collagen scaffolds repair critical-sized osteochondral defects under induced conditions of osteoarthritis in rat knee joints via inhibiting TGF-β-Smad1/5/8 signaling pathway
Source: Bioact Mater. 2024 Feb 16;35:416–28. doi: 10.1016/j.bioactmat.2024.02.008 (PMC10879694; doi:10.1016/j.bioactmat.2024.02.008)
Supplement: Multimedia component 1 [file mmc1.docx]

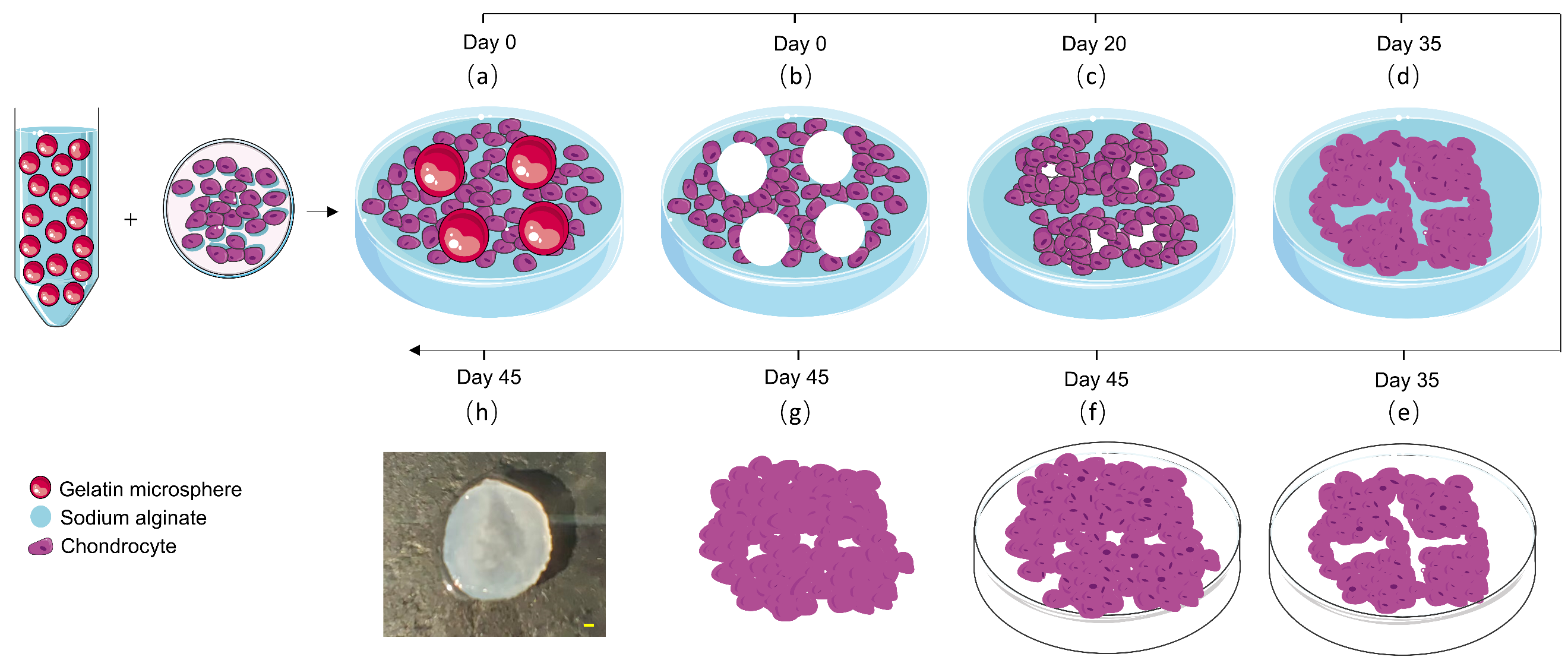


**Supplemental Figure 1.** Schematic illustration of Col2 scaffold fabrication. (a) Porcine chondrocytes are encapsulated with gelatin microspheres (porogen) within sodium alginate hydrogel. (b) Following the gelling of the alginate with a calcium chloride solution, the gelatin microspheres dissolve at 37℃, creating voids within the gel bulk. (c) The chondrocytes then proliferate within the alginate hydrogel and are guided to grow out of the gel phase, filling the voids and forming scattered pieces of pure microtissue. (d) These microtissues interact and secrete extracellular matrix (ECM) extensively, creating a complex interpenetrating network of ECM within the hydrogel. (e) The structural integrity of the construct remains intact after the removal of the alginate scaffold. (f) The scaffold-free construct is subsequently cultured in a medium for an additional 10 days to develop a living hyaline cartilage graft (LhCG). (g, h) LhCG is decellularized to produce a type II collagen based scaffold (dLhCG). Scale bar: 1mm.

**Supplemental Table 1**. The sample sizes for each experiment.

| **Experiments** | **Number of rats** |
| --- | --- |
| Histology and micro-CT |  |
| On Day 80 for 2mm OCDs | 1 |
| On Day 90 for 1.5mm OCDs (end point of observation) | 3 |
| On Day 100 for 2mm OCDs | 1 |
| On Day 150 for 2mm OCDs (end point of observation) | 3 |
| Negative control for 1.5mm OCD | 1 |
| Negative control for 2mm OCD | 1 |
| Biochemical assays |  |
| On Day 90 for 1.5mm OCDs | 3*3=9 |
| On Day 150 for 2mm OCDs | 2*3=6 |
| Biomechanical test |  |
| On Day 90 for 1.5mm OCDs | 3 |
| On Day 150 for 2mm OCDs | 3 |
| RNA sequencing |  |
| On Day 90 for 1.5mm OCDs | 2*3=6 |
| On Day 150 for 2mm OCDs | 2*3=6 |
| Mass spectrometry-based proteomics |  |
| On Day 90 for 1.5mm OCDs | 3*3=9 |
| On Day 150 for 2mm OCDs | 3*3=9 |
| Sum | 61 |


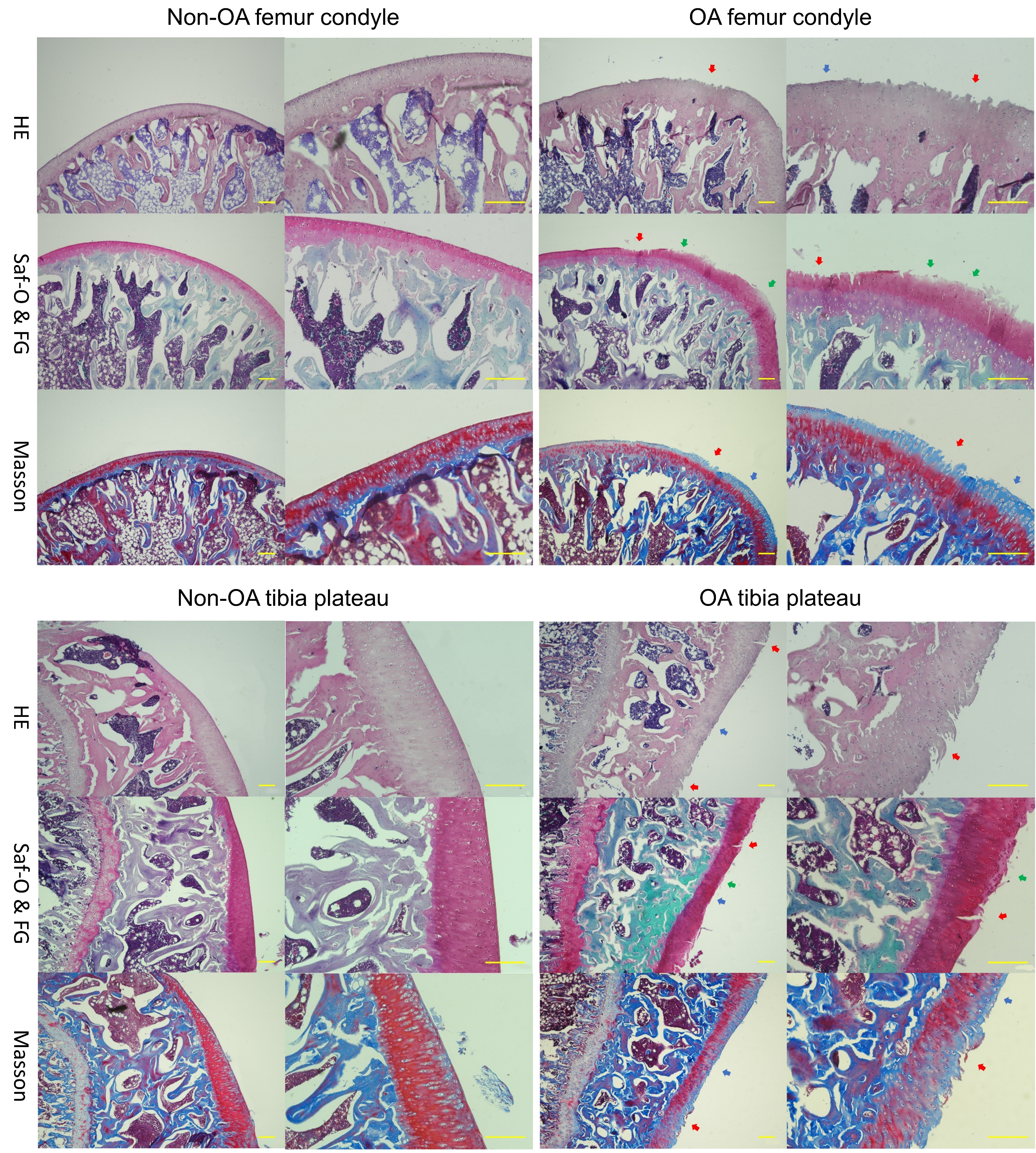


**Supplemental Figure 2.** The histological staining of critical-sized osteochondral defects on day 90 confirmed the induction of osteoarthritis. Red arrow, clefts at the superficial zone and extending to the deep zone; green arrow, a moderate decrease in staining; blue arrow, hypercellularity. Scale bar: 100 μm.


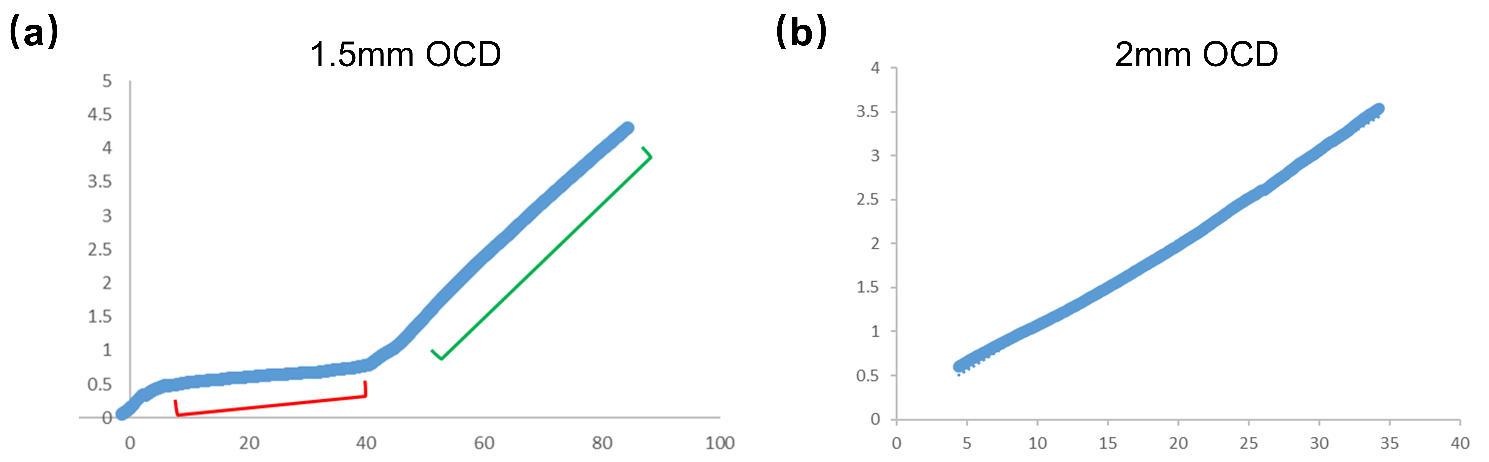


**Supplemental Figure 3.** The pattern of stress-strain curve varied depending on the size of osteochondral defects (OCDs). Notably, the stress-strain curve of the tissue from over critical-sized OCDs demonstrated a linear pattern (b), while that for critical-sized OCDs exhibited a discontinuous pattern (a). The initial segment of the discontinuous curve had a relatively small slope (red), which significantly increased in the latter segment (green).


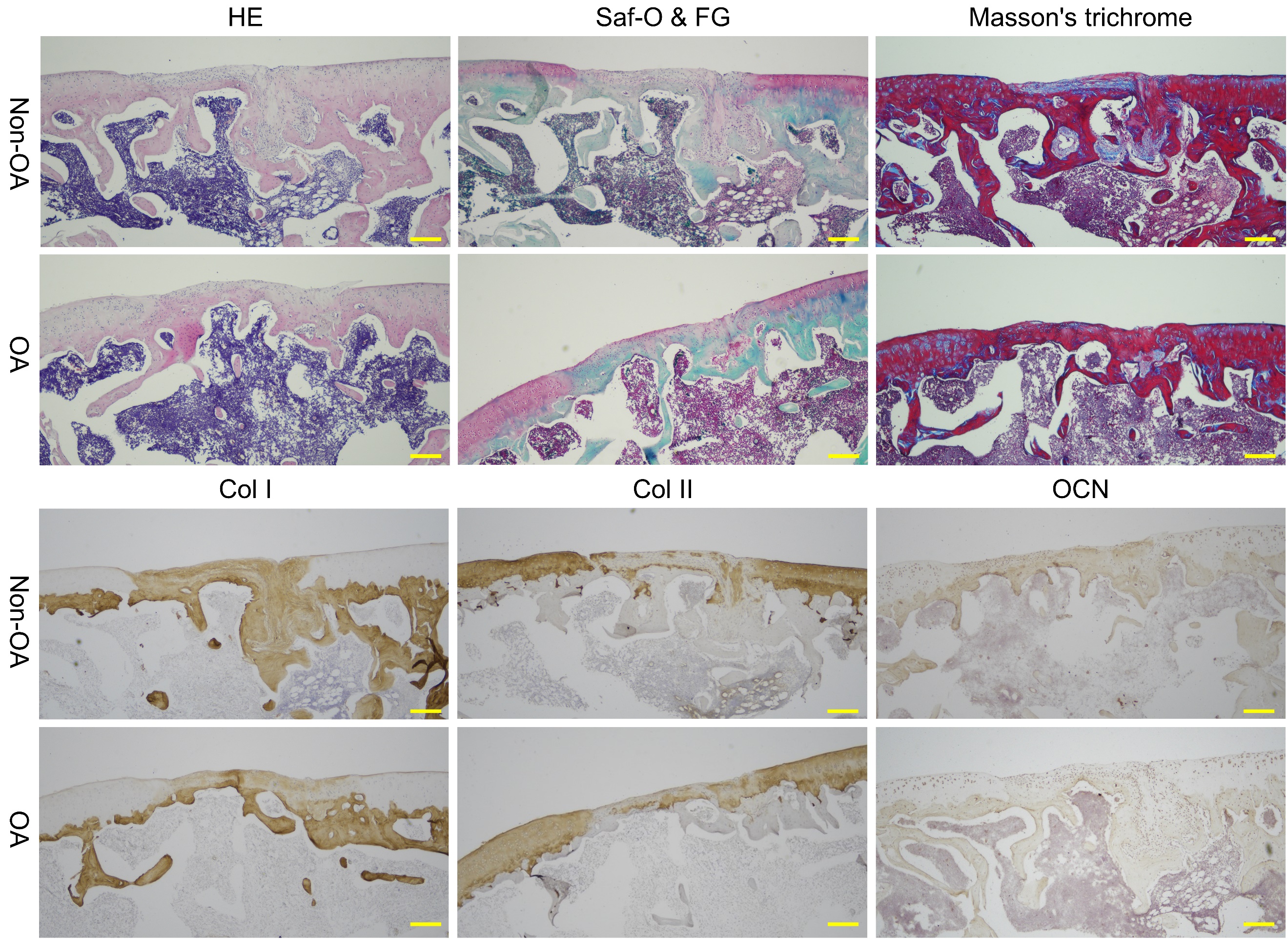


**Supplementary Figure 4.** Histological staining of over critical-sized osteochondral defects on day 80 revealed the persistence of the defect. Scale bar: 100 μm.


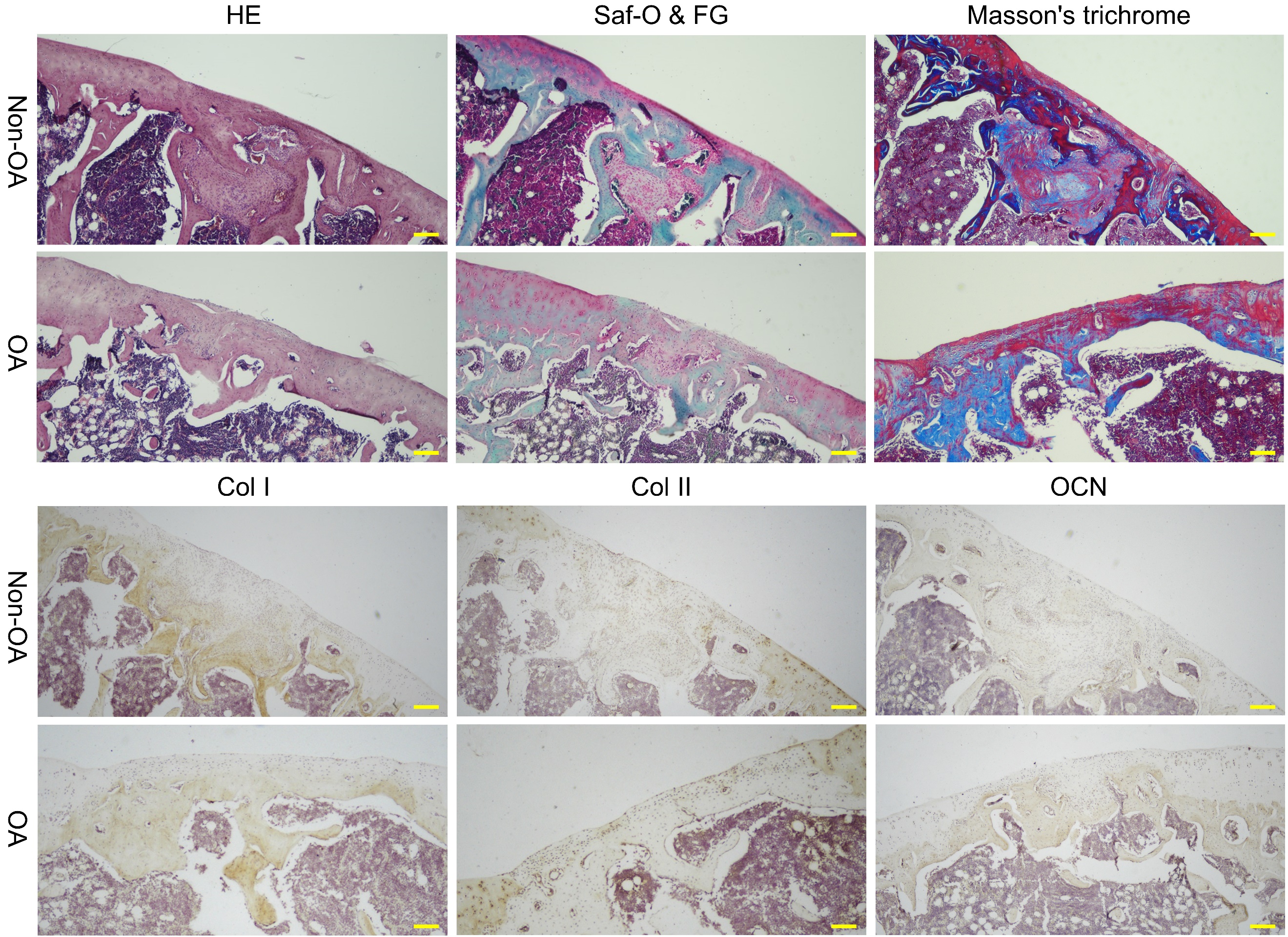


**Supplementary Figure 5.** Histological staining of over critical-sized osteochondral defects on day 100 revealed a partial repair of the osteochondral defects. Scale bar: 100 μm.


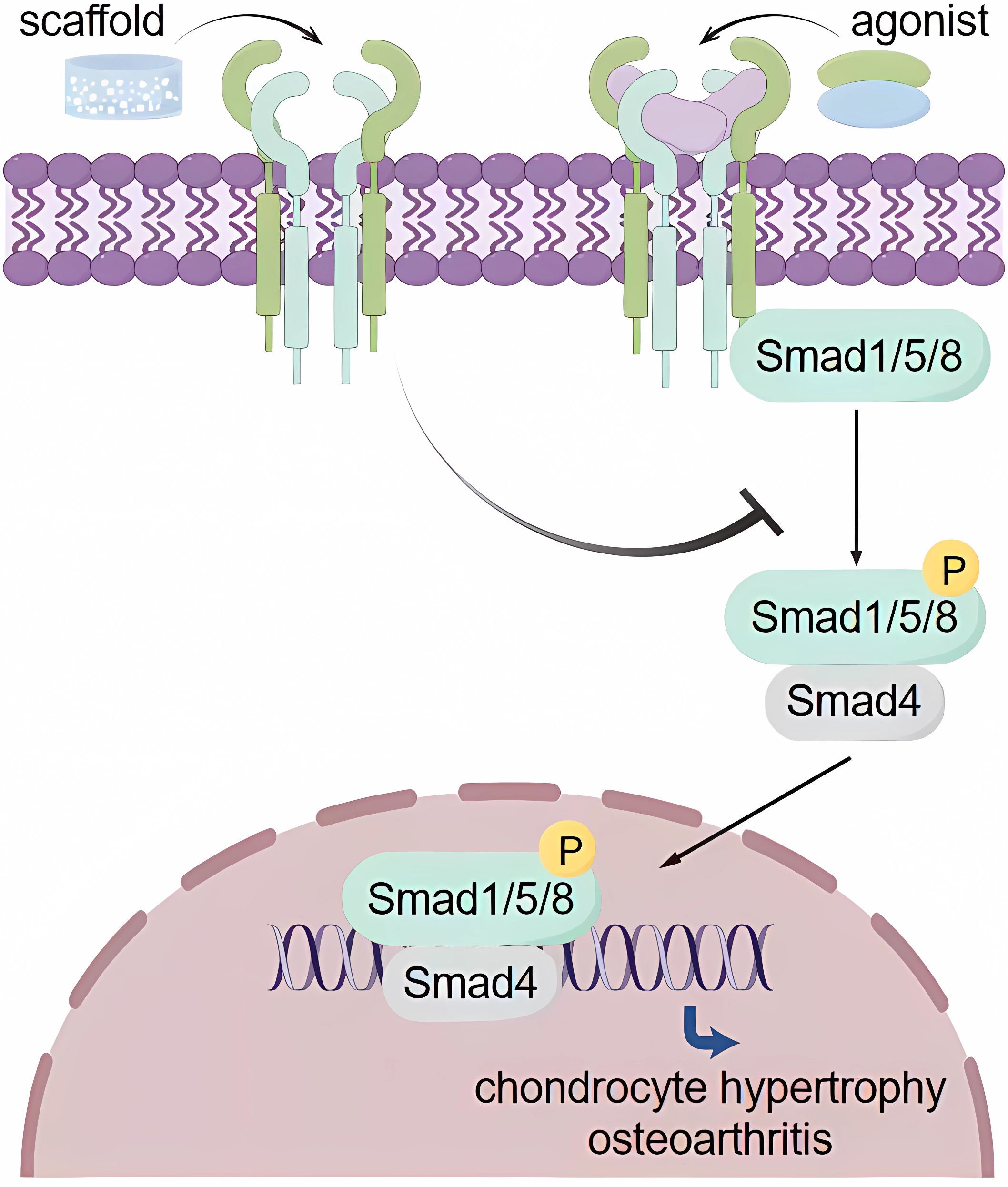


**Supplementary Figure 6**. Type II collagen scaffolds promote osteochondral defects regeneration via inhibiting TGF-β-Smad1/5/8 signaling pathway.


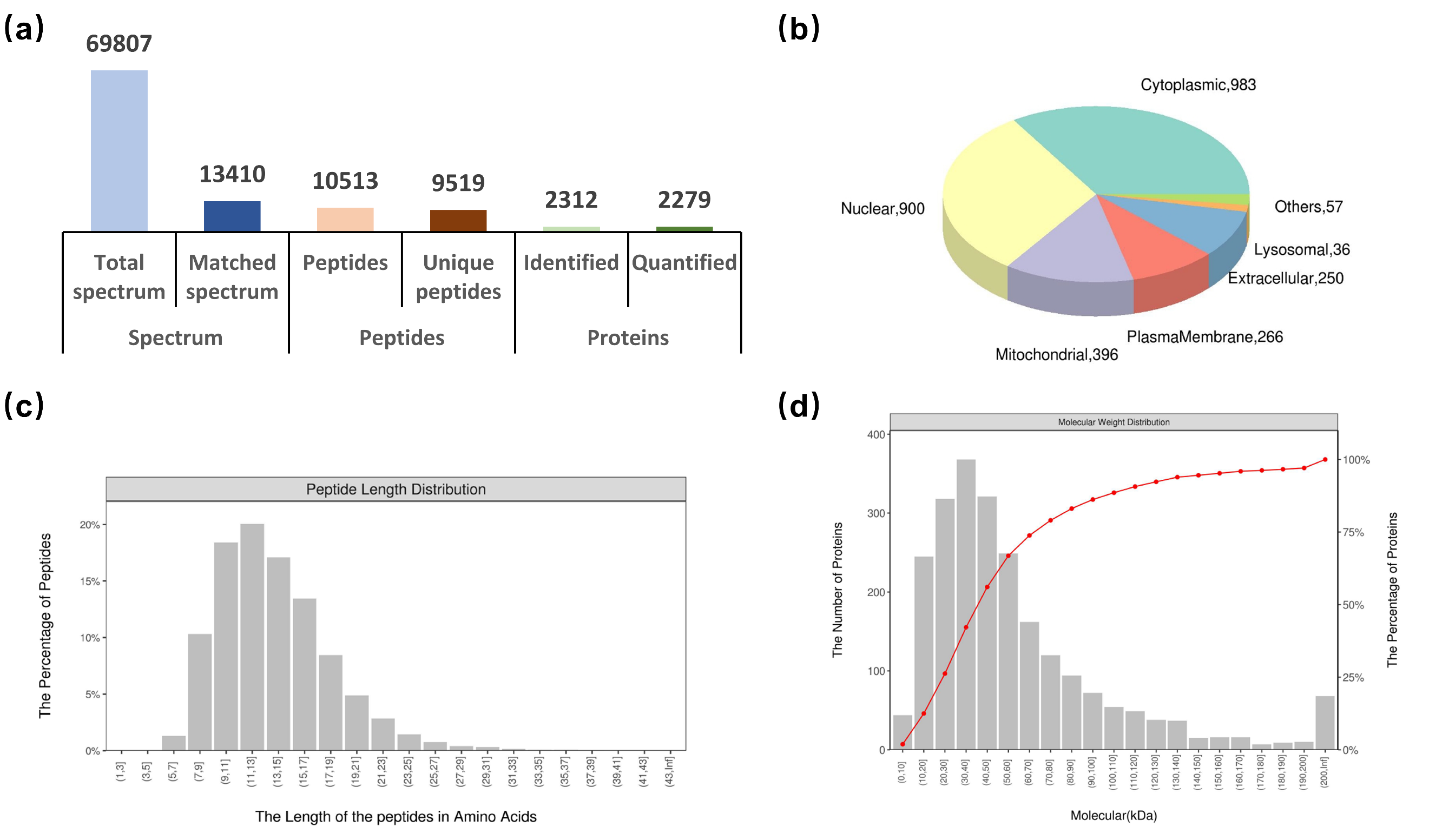


**Supplementary Figure 7**. Shotgun proteomics of type II collagen scaffolds. (a) Histogram for protein identification and quantitative results. (b) Pie chart for subcellular localization of identified proteins. (c) Length distribution of identified peptides. (d) Molecular mass distribution of the identified proteins
